# Risk of Cutibacterium acnes in Patients Undergoing Arthroscopic Rotator Cuff Repair Secondary to Unshaved Body Hair Within the Surgical Field

**DOI:** 10.7759/cureus.96741

**Published:** 2025-11-13

**Authors:** Sean McMillan, Alexander Blanca, Paul Favorito, Cory Lebowitz, Christopher McDowell, Manuel Pontes, Sundeep Saini, Elizabeth Ford

**Affiliations:** 1 Orthopedic Surgery, Virtua Health, Cherry Hill, USA; 2 Orthopedics, Broward Health Medical Center, Fort Lauderdale, USA; 3 Orthopedics, The Christ Hospital, Cincinnati, USA; 4 Orthopedic Surgery, Rowan University School of Osteopathic Medicine, Stratford, USA; 5 Orthopedics, Orthopaedics East & Sports Medicine Center, Greenville, USA; 6 Orthopedics, Rowan University, Glassboro, USA; 7 Orthopedics, Ocean Orthopedic Associates, Toms River, USA; 8 Orthopedic Surgery, Allegheny Health Network, Vineland, USA

**Keywords:** c. acnes, hair clipping, rotator cuff, shoulder infection, shoulder sport

## Abstract

Introduction

*Cutibacterium acnes *(*C. acnes*) infections in the setting of rotator cuff repair (RCR) can be devastating for a patient. Preoperative skin preparation prior to arthroscopic RCR typically consists of body hair clipping within the surgical field prior to prepping. We hypothesize that preoperative hair clipping will significantly reduce *C. acnes* colonization compared with unclipped controls.

Methods

All patients undergoing arthroscopic RCR from 2021 to 2025 were prospectively screened at a single institution for inclusion. Inclusion criterion included patients with skin hair present within the planned surgical field. The shoulder surgical field was divided into two sections. Section A underwent standard hair clipping. Section B was not clipped. The surgical field was prepped, and then one culture swab was taken from the shoulder section. All cultures were held for 21 days. Patients were followed for nine months to assess postoperative infection rates.

Results

The prevalence of *C. acnes* was 3.3% in the non-hair-clipped cohort and 0.0% in the hair-clipped cohort. The difference in positive cultures (shaved - unshaved) was -3.3%, 95% CI (-8.3, 0), p=0.018 (p<0.05). Bootstrap method analysis demonstrated a statistically significant decrease in infection rate in the shaved quadrants compared with the unshaved section. No postoperative infections were found at a minimum of nine months of follow-up.

Conclusion

Hair clipping within the surgical field for arthroscopic RCR resulted in a statistically significant decrease in *C. acnes *found on the skin.

## Introduction

Rotator cuff repair (RCR) is the most frequently studied arthroscopic shoulder procedure for skin and deep soft tissue infections [1]. Although the reported incidence of deep soft tissue infections following RCR is low, ranging from 0.3% to 1.9%, the true incidence may be higher due to underreporting [1]. Symptoms of infection include pain, stiffness, erythema, warmth, swelling, and fibrinous exudate at the shoulder, as well as systemic signs such as fever, fatigue, rotator cuff failure, and sepsis in severe or untreated cases [2]. Recognizing these manifestations in RCR patients and maintaining a high index of suspicion may help prevent unsatisfactory surgical outcomes and devastating complications.

Three of the most common causative pathogens associated with shoulder arthroscopy are *Cutibacterium acnes* (*C. acnes*), *Staphylococcus epidermidis*, and *Staphylococcus aureus* [3]. While intravenous antibiotics successfully treat most of these infections, delayed or missed diagnoses can lead to significant complications, including soft tissue destruction, adhesions, and loss of function.

*C. acnes*, formerly known as *Propionibacterium acnes* (*P. acnes*), has become a key focus of research among shoulder surgeons. This anaerobic, gram-positive bacillus is the most common pathogen implicated in deep soft tissue infections after both arthroscopic and open shoulder surgeries, typically located within the sebaceous glands of hair follicles and pores. Its propensity for colonizing the deep dermis of the head and shoulders, at rates higher than other body regions, makes it particularly concerning. Notably, females exhibit a consistently lower colonization rate than males, suggesting a potential link between body hair and increased bacterial growth [3].

To date, the literature is scant regarding the presence of body hair in the surgical field and the presence of pathogens or risk of infection following RCR. The aim of this study was to examine the potential risk of infection or bacterial seeding in patients undergoing arthroscopic RCR who require skin hair removal within the surgical field. We hypothesize that preoperative hair clipping will significantly reduce *C. acnes* colonization compared with unclipped controls.

## Materials and methods

Institutional Review Board (IRB) approval from Virtua Health, the primary investigator's (PI) home institution, was obtained before the study commenced. All patients undergoing arthroscopic RCR from 2021 to 2025 were prospectively screened at a single institution for inclusion by the PI. Inclusion and exclusion criteria for the study are listed in Table 1.

**Table 1:** Inclusion and exclusion criteria for C. acnes rotator cuff study The inclusion and exclusion criteria for participants considered for the *C. acnes* rotator cuff surgery study are listed above. Key components to study inclusion are the presence of body hair within the planned surgical field and the absence of active or previous skin infection on the affected shoulder.

Inclusion Criteria	Exclusion Criteria
Age over 18	Patients unable to have planned surgical skin prep due to a skin allergy
English-speaking	Planned concomitant open surgical procedure
Skin hair present within the planned surgical field	Active or history (within 3 months) of skin infection on the planned surgical site
Primary arthroscopic rotator cuff repair	Patients who refused to have their surgical field clipped
No previous shoulder surgery on the planned surgical side	-

**Figure 1:**
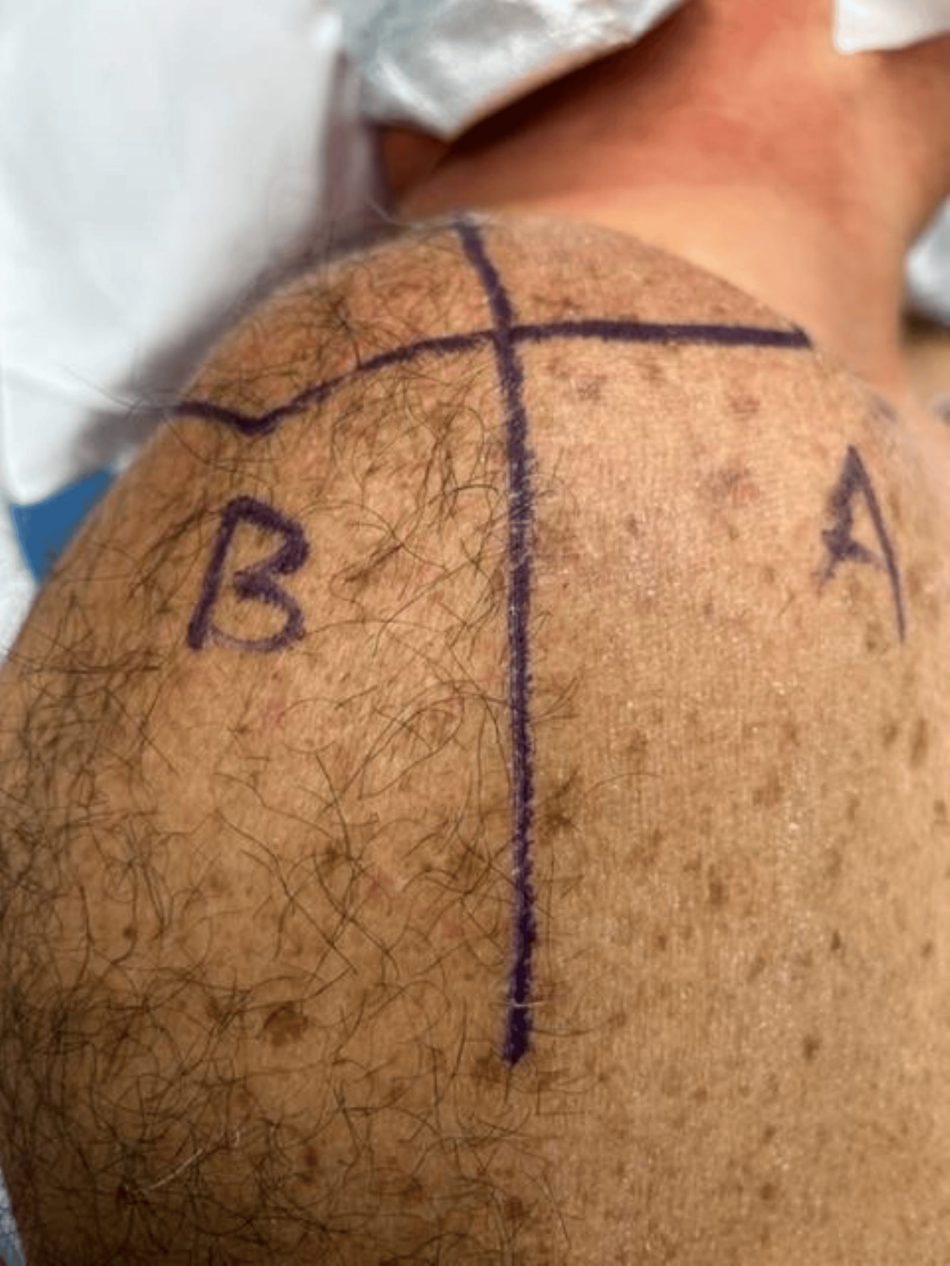
Surgical field image with shaved and unshaved sections The above image is of a right shoulder, viewed laterally, divided into two sections in preparation for culturing. Section A, defined as the anterior aspect of the shoulder from the coracoid to the mid-portion of the acromion laterally, has been surgically clipped to remove body hair. Section B, defined as the posterior aspect of the shoulder from the medial scapular border to the mid-portion of the acromion, has not been surgically clipped.

The shoulder surgical field was arbitrarily divided into two quadrants. "Section A" consisted of the region of skin within the surgical field between the coracoid anteriorly and moving laterally to the mid-portion of the acromion. "Section B" consisted of the region of skin within the surgical field between the mid-portion of the acromion extending posteriorly to the middle of the spine of the scapula (Figure 1).

Once the patient was positioned for the planned surgical procedure, Section A of the shoulder field underwent standard hair removal using surgical clippers. Section B was not clipped. Next, the shoulder surgical field was prepped using a 4% chlorhexidine scrub and 2% chlorhexidine gluconate/70% isopropyl alcohol paint (Chloraprep®). The skin prep was allowed to dry for three minutes, and then one culture swab was taken in Section A, and one culture swab was taken in Section B. Each section had both aerobic and anaerobic cultures performed. Each swab had five passes taken within a section longitudinally (cephalad to caudad) from the acromion down to the deltoid insertion. At the completion of the cultures, the remainder of the surgical field was clipped, and the shoulder was re-prepped for the planned surgical procedure.

Both sets of anaerobic and aerobic cultures from Section A and Section B were sent to microbiology and held for 21 days. At the end of 21 days, data were collected on the presence of any *C. acnes* growth or other bacteria. All patients were followed for nine months to determine if any postoperative infections occurred.

Statistical methods

The Newcombe score method was used to estimate the confidence interval and statistical significance of the rate difference in *C. acnes* presence between the unshaved and the shaved patient groups [4,5]. This confidence interval and the statistical tests were estimated using the R package ratesci [6]. The sample size required to achieve adequate power (0.80) to detect a significant difference (0.05) between the two groups was calculated using the R package exact2x2 [7].

In the present study, each patient had cultures performed on shaved and unshaved areas. As such, analyses of paired-sample proportions were conducted with IBM SPSS Statistics for Windows, Version 29 (Released 2023; IBM Corp., Armonk, New York). Confidence intervals and significance levels were estimated by bootstrap methods and the generation of 10,000 simulated samples [8].

## Results

A total of 374 patients were screened for inclusion from 2021 to 2025. Sixty patients met the inclusion criteria (16%). The prevalence of *C. acnes* was 3.3% (2/60) in the unclipped cohort and 0.0% (0/60) in the clipped cohort. The difference in positive cultures (shaved - unshaved) was -3.3%, 95% CI (-8.3, 0), p = 0.018 (p < 0.05). Bootstrap method analysis demonstrated a statistically significant decrease in infection rate in the shaved quadrants compared with the unshaved section. The results of the present study are listed in Table 2.

**Table 2:** Comparison of C. acnes colonization and postoperative infection rates between shaved and unshaved cohorts Table 2 presents the results of the current study, comparing colonization and postoperative infection rates of *C. acnes* between shaved and unshaved cohorts. The shaved cohort demonstrated a statistically significant reduction in *C. acnes* colonization compared with the unshaved cohort, although no symptomatic infections were observed in either group at a minimum of nine months of follow-up.

Outcome	Unclipped Cohort (n=60)	Clipped Cohort (n=60)	Comparison/Notes
Patients screened (2021–2025)	374 total screened	-	-
Patients included	60	60	-
*C. acnes* prevalence	3.3% (2/60)	0.0% (0/60)	Difference = -3.3%; 95% CI (-8.3, 0); p = 0.018
Other bacterial organisms	None detected	None detected	-
Symptomatic shoulder joint infection (≥9 months follow-up)	0%	0%	No difference
Statistical analysis	-	-	Bootstrap analysis confirmed a significant decrease in colonization in the shaved quadrants

The incidence of symptomatic shoulder joint infection after arthroscopic RCR was 0% in both the unshaved and shaved groups at a minimum of nine months of follow-up. No other bacterial organisms were found in either cohort.

## Discussion

This study aimed to investigate the correlation between the presence of body hair within the surgical field during arthroscopic RCR and the risk of bacterial contamination, specifically *C. acnes*. *C. acnes* infections in the setting of arthroscopic RCR can be devastating, with a reported 38% patient-reported unsatisfactory outcome eight years post-infection [2]. Our findings revealed no significant difference in bacterial growth rates between the hair-clipped and unclipped sections of the surgical field. These results challenge the hypothesis that residual body hair in the surgical field increases the risk of bacterial colonization and subsequent infection.

Marecek et al. previously reported on the bacterial burden of *C. acnes* related to axillary hair clipping [9]. Their findings showed no statistical difference in the risk of *C. acnes* between clipped and unclipped cultures taken from axillary hair. However, they noted a significantly greater bacterial burden in the clipped shoulder compared with the unclipped shoulder before preparation (P < .001), but not after preparation (P = .285). This is counter to the current study's findings, which noted the significance of *C. acnes* in the unclipped group after preparation. The authors chose to pursue the current study's hypothesis in complement to Marecek et al.'s work to examine a real-world clinical scenario. Specifically, during arthroscopic RCR, repair sutures often lie on the anterior, lateral, and posterior aspects of the skin during the procedure. Concern that sutures might lie on unclipped body hair during the repair prompted the hypothesis. The current study examined clipped and unclipped hair present within the surgical field, specifically during arthroscopic RCR, where sutures may be present that are implanted within the body. The differing locations of axillary hair versus surgical field hair are considered important.

Superficial skin colonization rate for *C. acnes* ranges from 42% to 73%, while deep tissue colonization rates are reported at 17-42%, even after routine surgical skin preparation and antibiotic prophylaxis [10,11]. Chuang et al. demonstrated that all patients with positive deep tissue cultures had positive superficial colonization prior to surgery, suggesting surgical instrument inoculation as a possible contamination source [11]. Additionally, Pauzenberger et al. found that preoperative antibiotic prophylaxis reduced overall postoperative infection rates from 1.54% to 0.28%, but this reduction did not significantly affect infections caused by *C. acnes* specifically [12].

Murray et al. demonstrated that chlorhexidine scrub was ineffective in reducing the burden of *C. acnes* prior to shoulder surgery (46% vs. 58%) [13]. Similarly, Chuang et al. observed that, despite preoperative chlorhexidine skin preparation and IV antibiotic prophylaxis, *C. acne* was cultured from deep tissues in 19.6% of patients who underwent arthroscopic shoulder surgery [11]. Singh et al. noted that decision-making aimed at decolonizing the skin for *C. acnes* must be balanced against the potential benefits of reduced deep tissue inoculation [14]. Numerous studies related to colonization have been conducted, specifically in the context of arthroplasty; however, little has been examined in the context of the current study [12,15-18]. The authors chose arthroscopic RCR for the procedure of choice in part due to the surgical repair sutures often being draped on the skin outside of the cannulas after implantation in the body. Indeed, Saltzman et al. noted that higher levels of *C. acnes* are associated with the region of the anterior and posterior arthroscopic portals [19]. We theorized that if the braided RCR repair sutures were lying on surgical field skin that was not clipped, there could be potential for seeding of the repair tissue. Despite the positive skin cultures in the unshaved group, the study did not yield symptomatic infections at nine months post-procedure.

Although our study demonstrated significance for the* C. acnes* burden in the unshaved group, limitations nonetheless exist. The patient population for this study was extremely narrow. The requirement of skin hair warranting removal essentially limited the study to male patients. This study included no female patients due to this parameter in our patient population. As noted, 374 patients over a period of four years who underwent arthroscopic RCR during the study enrollment were screen-failed due to a lack of hair within the surgical field that would typically require clipping. Second, bacterial colonization may exist at tissue depths beyond the reach of standard skin preparation methods. It is plausible that by not testing deeper skin layers, including the dermis and hypodermis, or the surgical tissues traversed before entering the joint space, potential sites of bacterial colonization may have been missed. However, investigating these deeper layers was beyond the scope of this study, as the purpose was to determine if validity exists for removing surgical skin hair within the arthroscopic field during RCR. Potentially revisiting the premise of this study in the context of an open surgical setting with a larger sample size may shed light on the potential ramifications of surgical hair clipping in terms of pathogen risk. Furthermore, in addition to the bacterial load, other factors may be involved in causing infection, which will require further investigation to come to a better understanding.

Surgical site hair removal prior to arthroscopic RCR is widely considered a customary practice. Despite this, there has yet to be a true clinical correlation between the results of this practice and the increased risk of skin colonization infection. The results of this study demonstrated a statistically significant difference between the two cohorts regarding *C. acnes* and surgical hair clipping. Future studies should focus on refining presurgical preparation techniques, employing larger sample sizes, and incorporating multiple culture swabs from various tissue depths. Such efforts could help identify effective methods to eradicate the risk of common skin pathogens, specifically *C. acnes*, and mitigate the risk of deep shoulder infections in arthroscopic RCR.

## Conclusions

This study demonstrated that preoperative shaving significantly reduced *C. acnes* colonization compared with unshaved controls. No symptomatic infections were observed in either group at a minimum of nine months of follow-up. These findings suggest that hair clipping may decrease bacterial burden at the surgical site, but its clinical impact on preventing postoperative shoulder infections remains uncertain.

## References

[1] Atesok K, MacDonald P, Leiter J, McRae S, Stranges G, Old J (2017). Postoperative deep shoulder infections following rotator cuff repair. World J Orthop.

[2] Athwal GS, Sperling JW, Rispoli DM, Cofield RH (2007). Deep infection after rotator cuff repair. J Shoulder Elbow Surg.

[3] Patel A, Calfee RP, Plante M, Fischer SA, Green A (2009). Propionibacterium acnes colonization of the human shoulder. J Shoulder Elbow Surg.

[4] Alyea E, Gaston T, Austin LS, Wowkanech C, Cypel B, Pontes M, Williams G (2019). The effectiveness of aspirin for venous thromboembolism prophylaxis for patients undergoing arthroscopic rotator cuff repair. Orthopedics.

[5] Newcombe RG (1998). Interval estimation for the difference between independent proportions: comparison of eleven methods. Stat Med.

[6] Laud PJ (2019). ratesci: confidence intervals for comparisons of binomial or Poisson rates. https://cran.r-project.org/.

[7] Fay MP (2010). Confidence intervals that match Fisher's exact or Blaker's exact tests. Biostatistics.

[8] Zaniletti I, Larson DR, Lewallen DG, Berry DJ, Maradit Kremers H (2023). How to develop and validate prediction models for orthopedic outcomes. J Arthroplasty.

[9] Marecek GS, Weatherford BM, Fuller EB, Saltzman MD (2015). The effect of axillary hair on surgical antisepsis around the shoulder. J Shoulder Elbow Surg.

[10] Foster AL, Cutbush K, Ezure Y, Schuetz MA, Crawford R, Paterson DL (2021). Cutibacterium acnes in shoulder surgery: a scoping review of strategies for prevention, diagnosis, and treatment. J Shoulder Elbow Surg.

[11] Chuang MJ, Jancosko JJ, Mendoza V, Nottage WM (2015). The incidence of Propionibacterium acnes in shoulder arthroscopy. Arthroscopy.

[12] Pauzenberger L, Grieb A, Hexel M, Laky B, Anderl W, Heuberer P (2017). Infections following arthroscopic rotator cuff repair: incidence, risk factors, and prophylaxis. Knee Surg Sports Traumatol Arthrosc.

[13] Murray MR, Saltzman MD, Gryzlo SM, Terry MA, Woodward CC, Nuber GW (2011). Efficacy of preoperative home use of 2% chlorhexidine gluconate cloth before shoulder surgery. J Shoulder Elbow Surg.

[14] Singh AM, Sethi PM, Romeo AA, Anakwenze OA, Abboud JA, Namdari S (2020). Strategies to decolonize the shoulder of Cutibacterium acnes: a review of the literature. J Shoulder Elbow Surg.

[15] Iqbal A, Javaid MA, Sohail M, Khan F (2024). A literature review of Cutibacterium acnes: from skin commensal to pathogen in shoulder surgery. Cureus.

[16] Kolakowski L, Lai JK, Duvall GT (2018). Neer Award 2018: benzoyl peroxide effectively decreases preoperative Cutibacterium acnes shoulder burden: a prospective randomized controlled trial. J Shoulder Elbow Surg.

[17] Phadnis J, Gordon D, Krishnan J, Bain GI (2016). Frequent isolation of Propionibacterium acnes from the shoulder dermis despite skin preparation and prophylactic antibiotics. J Shoulder Elbow Surg.

[18] Sabetta JR, Rana VP, Vadasdi KB, Greene RT, Cunningham JG, Miller SR, Sethi PM (2015). Efficacy of topical benzoyl peroxide on the reduction of Propionibacterium acnes during shoulder surgery. J Shoulder Elbow Surg.

[19] Saltzman MD, Nuber GW, Gryzlo SM, Marecek GS, Koh JL (2009). Efficacy of surgical preparation solutions in shoulder surgery. J Bone Joint Surg Am.

